# IFN-γ and IL-5 whole blood response directed against mycolactone polyketide synthase domains in patients with *Mycobacterium ulcerans* infection

**DOI:** 10.7717/peerj.5294

**Published:** 2018-07-31

**Authors:** Aloysius D. Loglo, Michael Frimpong, Mabel Sarpong Duah, Fred Sarfo, Francisca N. Sarpong, Bernadette Agbavor, Justice K. Boakye-Appiah, Kabiru M. Abass, Mathias Dongyele, Margaret Frempong, Sacha Pidot, Mark Wansbrough-Jones, Timothy P. Stinear, Virginie Roupie, Kris Huygen, Richard O. Phillips

**Affiliations:** 1 Kumasi Centre for Collaborative Research in Tropical Medicine, Kwame Nkrumah University of Science and Technology, Kumasi, Ghana; 2 School of Medical Sciences, Kwame Nkrumah University of Science and Technology, Kumasi, Ghana; 3 Institute of Infection and Immunity, St George’s University of London, London, UK; 4 Agogo Presbyterian Hospital, Agogo, Ghana; 5 Tepa Government Hospital, Tepa, Ghana; 6 Department of Microbiology and Immunology, Doherty Institute for Infection and Immunity, University of Melbourne, Melbourne, VIC, Australia; 7 Service Immunology, Scientific Institute of Public Health, Brussels, Belgium

**Keywords:** *Mycobacterium ulcerans*, Immune response, Polyketide synthase domains, Buruli ulcer

## Abstract

**Background:**

Buruli ulcer is a disease of the skin and soft tissues caused by infection with a slow growing pathogen, *Mycobacterium ulcerans*. A vaccine for this disease is not available but *M. ulcerans* possesses a giant plasmid pMUM001 that harbours the polyketide synthase (PKS) genes encoding a multi-enzyme complex needed for the production of its unique lipid toxin called mycolactone, which is central to the pathogenesis of Buruli ulcer. We have studied the immunogenicity of enzymatic domains in humans with *M. ulcerans* disease, their contacts, as well as non-endemic areas controls.

**Methods:**

Between March 2013 and August 2015, heparinized whole blood was obtained from patients confirmed with Buruli ulcer. The blood samples were diluted 1 in 10 in Roswell Park Memorial Institute (RPMI) medium and incubated for 5 days with recombinant mycolactone PKS domains and mycolyltransferase antigen 85A (Ag85A). Blood samples were obtained before and at completion of antibiotic treatment for 8 weeks and again 8 weeks after completion of treatment. Supernatants were assayed for interferon-γ (IFN-γ) and interleukin-5 (IL-5) by enzyme-linked immunosorbent assay. Responses were compared with those of contacts and non-endemic controls.

**Results:**

More than 80% of patients and contacts from endemic areas produced IFN-γ in response to all the antigens except acyl carrier protein type 3 (ACP3) to which only 47% of active Buruli ulcer cases and 71% of contacts responded. The highest proportion of responders in cases and contacts was to load module ketosynthase domain (Ksalt) (100%) and enoylreductase (100%). Lower IL-5 responses were induced in a smaller proportion of patients ranging from 54% after ketoreductase type B stimulation to only 21% with ketosynthase type C (KS C). Among endemic area contacts, the, highest proportion was 73% responding to KS C and the lowest was 40% responding to acyltransferase with acetate specificity type 2. Contacts of Buruli ulcer patients produced significantly higher IFN-γ and IL-5 responses compared with those of patients to PKS domain antigens and to mycolyltransferase Ag85A of *M. ulcerans*. There was low or no response to all the antigens in non-endemic areas controls. IFN-γ and IL-5 responses of patients improved after treatment when compared to baseline results.

**Discussion:**

The major response to PKS antigen stimulation was IFN-γ and the strongest responses were observed in healthy contacts of patients living in areas endemic for Buruli ulcer. Patients elicited lower responses than healthy contacts, possibly due to the immunosuppressive effect of mycolactone, but the responses were enhanced after antibiotic treatment. A vaccine made up of the most immunogenic PKS domains combined with the mycolyltransferase Ag85A warrants further investigation.

## Introduction

Buruli ulcer is a disease of the skin and soft tissues caused by infection with a slow growing pathogen, *Mycobacterium ulcerans* (Demangel, Stinear & Cole, 2009). A large proportion of the cases are reported by 33 countries from tropical, subtropical and temperate climates in Africa, South America and the Western pacific regions respectively (WHO, 2015). Globally, 5,000–6,000 cases are reported annually from 15 out of 33 countries (WHO, 2015). Children between the age of 5 and 15 years account for 48% of African cases but only 10% from Australia, and 19% from Japan (WHO). The majority of cases reported from sub-Saharan Africa are from poor rural communities. The disease usually manifests as a painless nodule, a firm plaque, or an ulcer with characteristic undermined edges and more severe lesions may be associated with surrounding oedema (WHO, 2015). Treatment has shifted from surgery to antibiotic therapy with the combination of oral rifampicin and either intramuscular streptomycin or oral clarithromycin for 56 days. Although there is a strong association with living near stagnant or slow flowing water bodies, the mode of transmission of the infection remains unknown (Portaels et al., 1999; Merritt et al., 2010; Marion et al., 2010) and development of a protective vaccine is highly desirable.

Currently, there is no vaccine for *M. ulcerans* disease. In early studies *M. bovis* BCG vaccine appeared to be protective for 6 months after vaccination but thereafter its effect waned. However, these findings were not conclusive because the disease incidence declined in the study area (Smith et al., 1976; Uganda Buruli Group, 1969; Huygen, 2003; Nackers et al., 2006; Portaels et al., 2004; Tanghe et al., 2007). Recently a retrospective study failed to demonstrate any protection associated with the presence of BCG scars and there was no apparent protection from the severe forms of disease (Phillips et al., 2015). However, evidence has been presented that BCG protects against the osteomyelitis complicating *M. ulcerans* infection. A study looking at the protective efficacy of a DNA vaccine encoding antigen 85A (Ag85A) from *M. bovis* BCG showed that it could significantly reduce the bacterial load in footpads of mice challenged with *M. ulcerans* (Tanghe et al., 2001). DNA vaccination with *M. ulcerans* heat shock protein-65 (HSP-65) also conferred some protection against *M. ulcerans* in mice (Coutanceau et al., 2006) but although HSP-65 protein was immunogenic, it may not be a good vaccine candidate since it shares homology with human HSP-60.

*Mycobacterium ulcerans* possesses a giant plasmid PMUM001 that encodes polyketide synthases (PKSs) needed for the production of its unique lipid toxin called mycolactone (Stinear et al., 2004; Porter et al., 2013). This macrolide toxin is central to the pathogenesis of Buruli ulcer and it has cytotoxic and immunosuppressive properties but it is poorly immunogenic. Therefore, in the efforts to develop a vaccine, one approach has been to target the enzymatic domains that play a key role in synthesis of mycolactone (Porter et al., 2013). A recent study in mice showed that *E. coli* derived recombinant proteins generated by plasmid DNA encoding mycolactone PKS domains were immunogenic providing modest protection (Roupie et al., 2014).

In this study, IFN-γ was selected as a T-helper 1 (Th1) marker while Interleukin-5 (IL-5) was measured for T-helper 2 (Th2) responses. The best defence against mycobacterial infections is driven by the production of IFN-γ by helper T-cells. Activated IFN-γ can increase the ability of macrophages to kill intracellular mycobacteria by nitric oxide induced apoptosis (Bibert et al., 2017). IL-5 is a cytokine that acts as a growth and differentiation factor for both B-cells and eosinophils. The encoded cytokine plays a major in the regulation of eosinophils formation, maturation, recruitment and survival (Clutterbuck, Hirst & Sanderson, 1989; Sanderson, 1992).

The aim of the present study was to understand the immunogenicity of similar antigens in humans with *M. ulcerans* disease and their contacts as well as in control participants from non-endemic areas by the induction of antigen specific cellular immune response.

## Materials and Methods

### Study participants and design

Between March 2013 and August 2015, consecutive patients with clinically suspected Buruli ulcer presenting at clinics in Agogo Presbyterian Hospital in the Ashanti-Akim-North district, Tepa Government Hospital in the Ahafo-Ano-district, Nkawie-Toase Government Hospital of the Atwima Nwabiagya district in the Ashanti Region and Dunkwa Government Hospital in the Upper Denkyira East district in the Central Region of Ghana, were asked to participate in the study. Concurrently, age matched contacts of patients from the endemic districts and non-endemic controls living in Kumasi where Buruli ulcer is not endemic were recruited. Patients and controls were only recruited after oral and written consent had been secured. Patients were enrolled if they met the WHO clinical case definition of *M. ulcerans* disease (WHO, 2015) and were included if the clinical diagnosis of *M. ulcerans* disease was subsequently confirmed by a positive IS2404 PCR.

Patients were excluded when they were aged below 5 years, were unwilling to give informed consent or already on treatment. Ethical approval for this study was obtained from the Committee on Human Research, Publications and Ethics (CHRPE/AP/229/12), School of Medical Sciences, Kwame Nkrumah University of Science and Technology, Kumasi, Ghana. An endemic control or contact was an age matched healthy relative, spouse or neighbour permanently residing in the same community. Endemic controls were verified to have no past or current diagnosis of Buruli ulcer. Participants who did not visit or reside in a Buruli endemic area were considered as non-endemic controls.

For patients with Buruli ulcer, basic demographic data including age, sex, lesion form, lesion site and category were recorded on standard WHO BU01 forms (WHO, 2018). Swabs from ulcers and fine needle aspirates from pre-ulcerative lesions were obtained for *M. ulcerans* IS2404 PCR and also combined with the *M. ulcerans* 16S rRNA as previously described (Phillips et al., 2009; Sarpong-Duah et al., 2017; Sarfo et al., 2010). Patients were treated with combination oral rifampicin 10 mg/kg and intramuscular streptomycin 15 mg/kg daily for 8 weeks or oral clarithromycin. This was administered at village health posts under direct observation with fortnightly visits to participating hospitals for clinical review and assessment of compliance to antibiotic therapy. After antibiotic therapy patients were followed up monthly for one year.

Heparinized blood samples were obtained for immune response assessment before and at completion of antibiotic treatment at week 8 and again 8 weeks after completion of treatment.

### Antigens

Bacterial expression vectors pET-DEST42, encoding the genes of 11 mycolactone PKS domains, namely, acyl carrier protein (ACP) types 2 and 3, acyltransferase with acetate specificity type 1 and 2 (ATac1, ATac2), acyltransferase with propionate specificity (ATp), enoylreductase (ER), ketoreductase type A and B (KR A, KR B), ketosynthase type C (KS C), load module ketosynthase domain (Ksalt) and dehydratase (DH), (all as C-terminally Histidine-tagged proteins), were constructed at the University of Melbourne, Australia, sent to Brussels and used for transformation and selection in *E. coli* BL-21. Following induction with Isopropyl-β-D-thioglactopyranoside (IPTG) for 2–4 h, cells were lysed and recombinant proteins were purified according to standard protocol on immobilized metal affinity chromatography using gravity flow. Recombinant Ag85A protein from *M. ulcerans* (MUL 4987) was kindly given to us by Dr. G. Pluschke (Swiss Tropical and Public Health Institute, Basel, Switzerland) (Roupie et al., 2014). All antigens were used at a final concentration of five μg/ml based on experiences from an earlier study (Phillips et al., 2006).

Positive controls were one μg/ml phorbol 1-myristate 1-acetate plus ionomycin (PMA + Io) and five μg/ml lipopolysaccharide (Sigma-Aldrich, Dorset, UK) while medium alone was the negative control.

### Whole blood antigen stimulation

A total of six ml of venous blood was taken in sodium heparin Vacutainer tubes (Becton Dickinson, Oxford, UK) at baseline, week 8 and week 16 and transported to the laboratory for diluted whole assay within 2 h of sampling. The whole-blood assay was performed as described previously (Sarfo et al., 2009). Briefly, whole blood was diluted 1 in 10 in Roswell Park Memorial Institute medium supplemented with penicillin (100 IU/ml) and streptomycin (100 g/ml) (Sigma-Aldrich, Darmstadt, Germany) in sterile 50 ml Falcon tubes, mixed gently and distributed, one ml per well into 24-well plates (Nunclon, Roskilde, Denmark). A total five μg/ml of PMA, recombinant ACP2, ACP3, Atac1, Atac2, ATp, ER, KR A, KR B, KS C, Ksalt, DH, Ag85Aulc and no stimulant was pipetted into each well, after which the culture plates were gently swirled 10 times clockwise and anticlockwise. Culture plates were incubated at 37 °C in 5% CO_2_ for 5 days. Supernatants (300–500 μl per well) were stored at −70 °C for interferon-γ (IFN-γ) and IL-5 assays.

### Enzyme linked immunosorbent assay

Human IFN-γ and IL-5 concentrations were determined using OptEIA ELISA kits in accordance with the protocol provided by manufacturer (BD Biosciences, Pharmingen, San Diego, CA, USA). Optical densities at 450–620 nm were measured with an ELISA plate reader (Sunrise Tecan, Salzburg, Austria) with xread plus version 4.30 software. IFN-γ recombinant cytokine (300 to 4.7 pg/ml; BD Biosciences, Pharmingen, San Diego, CA, USA) was used for the standard curve. IL-5 recombinant cytokine (BD Biosciences, Pharmingen, San Diego, CA, USA) was used for the standard curve. The lower detection limits were 4.7 pg/ml for IFN-γ and 7.8 pg/ml for IL-5. A positive cytokine measurement in the unstimulated culture supernatants, if detected, was subtracted from measurements in the test wells for IFN-γ and IL-5. In the analysis, a cut off of 37 pg/ml was used for both IFN-γ and IL-5 to define a responder status since none of the subjects produced more than 37 pg/ml in unstimulated cultures.

### Combined 16S rRNA reverse transcriptase/IS2404 qPCR viability assay

Fine needle aspirates and swab samples were transported from study site to the KCCR stabilized in 500 μl RNA protect (Qiagen, Manchester, UK) for the *M. ulcerans* combined 16S rRNA reverse transcriptase/IS2404 qPCR viability assay as described elsewhere (Sarpong-Duah et al., 2017). Briefly, whole transcriptome RNA and whole genome DNA were extracted from the same clinical sample. The RNA and DNA isolation was carried out using the AllPrep DNA/RNA Micro kit (Qiagen, Manchester, UK) as previously described (Beissner et al., 2012; Sarpong-Duah et al., 2017). A total of 12 μl RNA extracts were immediately reverse transcribed (Sarpong-Duah et al., 2017). The cDNA was then subjected to 16S rRNA qPCR and DNA to IS2404 qPCR to increase the specificity for *M. ulcerans*. Quantitative PCR of IS2404 (DNA), and 16S rRNA (cDNA) targets were carried out at 95 °C for 15 min, and then 40 cycles of 95 °C for 15 s and 60 °C for 60 s in a Bio-Rad CFX 96 real time PCR detection system (Bio-Rad, Singapore).

### Statistical analysis

Results were analysed with GraphPad Prism 6 software (GraphPad Software Inc., La Jolla, CA, USA) and a standard (best-fit) curve was plotted. Descriptive results of cytokines were expressed as medians and ranges. Medians for participants at the various time points of the study were compared using the Mann–Whitney *U*-test; *P* < 0.05 were considered significant. IFN-γ responses of patients with positive 16S rRNA reverse transcriptase/IS2404 qPCR signal suggestive of viable *M. ulcerans* at week 8 were noted.

## Results

### Participant characteristics

Table 1 shows the characteristics of 24 participants with active Buruli ulcer disease, 30 endemic area patient contacts and 11 non-endemic area controls. Mean and standard deviation (±SD) of age was 20 (±17) years for patients with active lesions, 21 (±13) years for contacts and 24 (±4) years for controls from non-endemic regions. One-way analysis of variance showed no significant difference in the ages of the participants (*P* = 0.6957). There were 11 patients with non-ulcerative forms (four nodules and seven plaques) and 13 patients with ulcers.

**Table 1:** Characteristics of study participants.

Number of participants	Active Buruli ulcer *n* = 24	Contacts* *n* = 30	Non-endemic controls** *n* = 11
Age			
Mean (±SD)	20 (±17)	21 (±13)	24 (±4)
Sex			
Male to female ratio	13:11	14:16	7:4
Lesion form (%)			
Nodule	4 (17)	N/A	N/A
Plaque	7 (29)	N/A	N/A
Ulcer	13 (54)	N/A	N/A
Lesion category (%)			
I	10 (42)	N/A	N/A
II	11 (46)	N/A	N/A
III	3 (12)	N/A	N/A

**Notes:**

N/A: not applicable. One-way analysis of variance test showed no differences in age distribution between the study groups (*P* = 0.6957).

* Contacts: family members of Buruli ulcer patients living in the endemic area.

** Non-endemic controls were resident in Buruli ulcer non-endemic areas.

### IFN-γ and IL-5 responses to 11 recombinant PKS domain antigens or Ag85A in Buruli ulcer patients, contacts and non-endemic area controls

**Figure 1:**
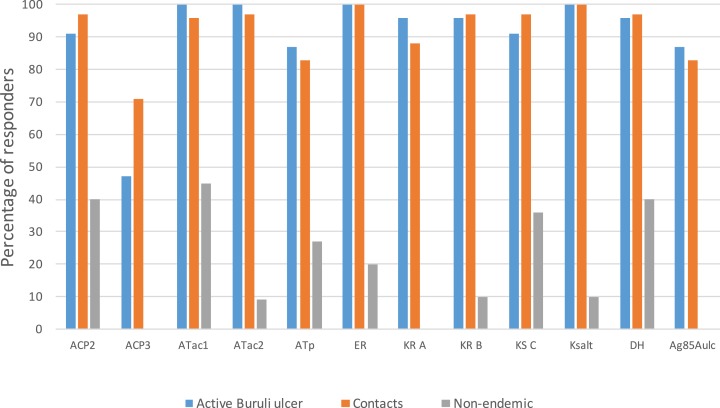
IFN-γ responses to a panel of polyketide synthase domains in 5 day diluted whole blood culture supernatants of *M. ulcerans* disease patients, healthy contacts and healthy non-endemic controls. A cut off of 37 pg/ml of IFN-γ was used to define responder status. Each bar represent percentage (%) of responders of a participant group to a specific PKS domain antigen.

Using a cut off of 37 pg/ml of IFN-γ to define a responder status, more than 80% of patients and contacts from endemic areas responded to all the antigens except ACP3 to which only 47% of active Buruli ulcer cases and 71% of contacts responded. The highest proportion of responders in both patients and endemic area controls was to Ksalt (100%) and ER (100%). Overall, a higher proportion of contacts responded with higher IFN-γ responses than the Buruli ulcer cases to all the PKS domain antigens and to DNA encoding mycolyltransferase Ag85A of *M. ulcerans* (Ag85Aulc). There was low or no response to any of the antigens in all except one of the non-endemic area controls (Fig. 1).

**Figure 2:**
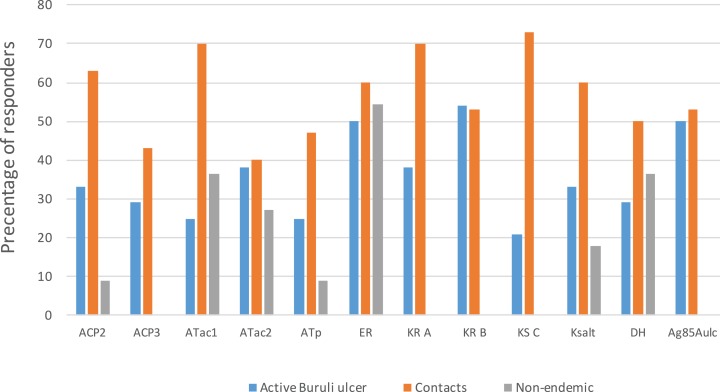
IL-5 responses to a panel of polyketide synthase domains in 5 day diluted whole blood culture supernatants of *M. ulcerans* disease patients, healthy contacts and healthy non-endemic controls. A cut off of 37 pg/ml of IL-5 was used to define responder status. Each bar represent percentage (%) of responders of a participant group to a specific PKS domain antigen.

With a cut off of 37 pg/ml for IL-5, responses of cases and endemic controls were modest compared to those for IFN-γ. The highest proportion of responders among BU cases was to KR B (54%) and the lowest to KS C (21%). Among endemic area contacts the highest proportion was to KS C (73%) and the lowest to Atac2 (40%). There were low or nil responses in non-endemic controls apart from 6/11 (55%) responding to ER (Fig. 2).

### Comparison of IFN-γ and IL-5 responses between *M. ulcerans* disease cases, healthy contacts and healthy non-endemic controls

**Figure 3:**
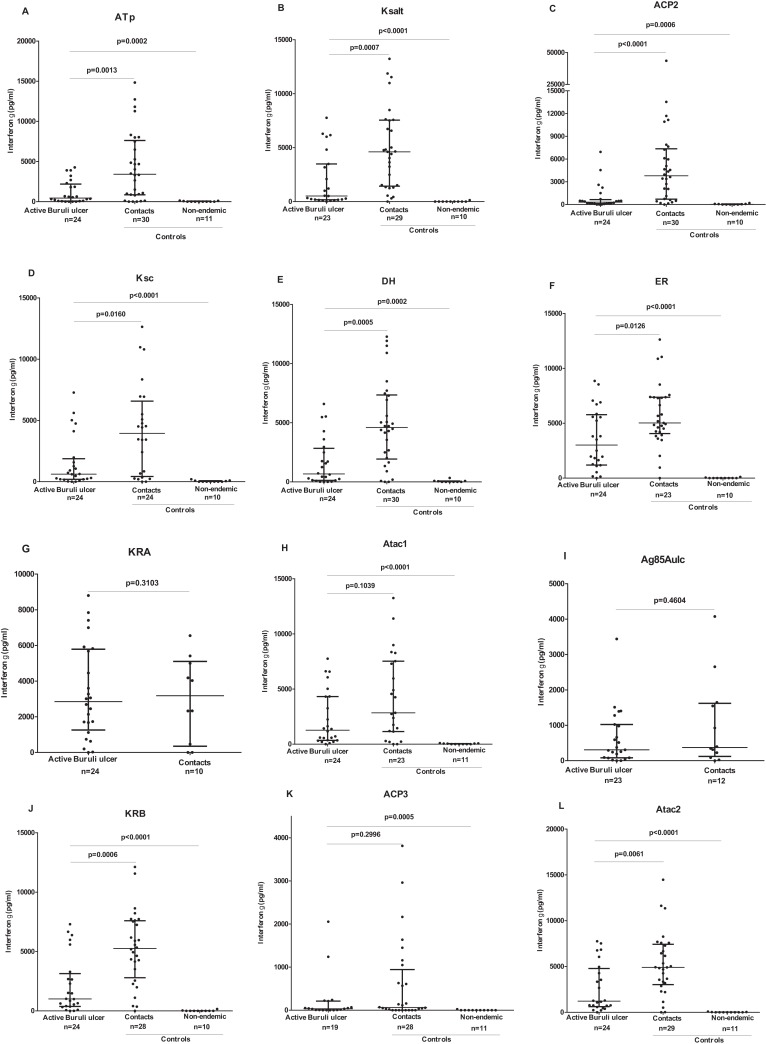
IFN-γ responses to a panel of polyketide synthase domain antigens in 5 day diluted whole blood culture supernatants of *Mycobacterium ulcerans* disease patients, healthy endemic controls and healthy non-endemic controls. IFN-γ responses are shown for ATp (A), Ksalt (B), ACP2 (C), Ksc (D), DH (E), ER (F), KRA (G), Atac1 (H), Ag85Aulc (I), KRB (J), ACP3 (K) and Atac2 (L). Each dot represents the response of one study participant. The horizontal lines represent the median and Interquartile range for each group. Medians for groups were compared using Mann-Whitney *U-test*.

**Figure 4:**
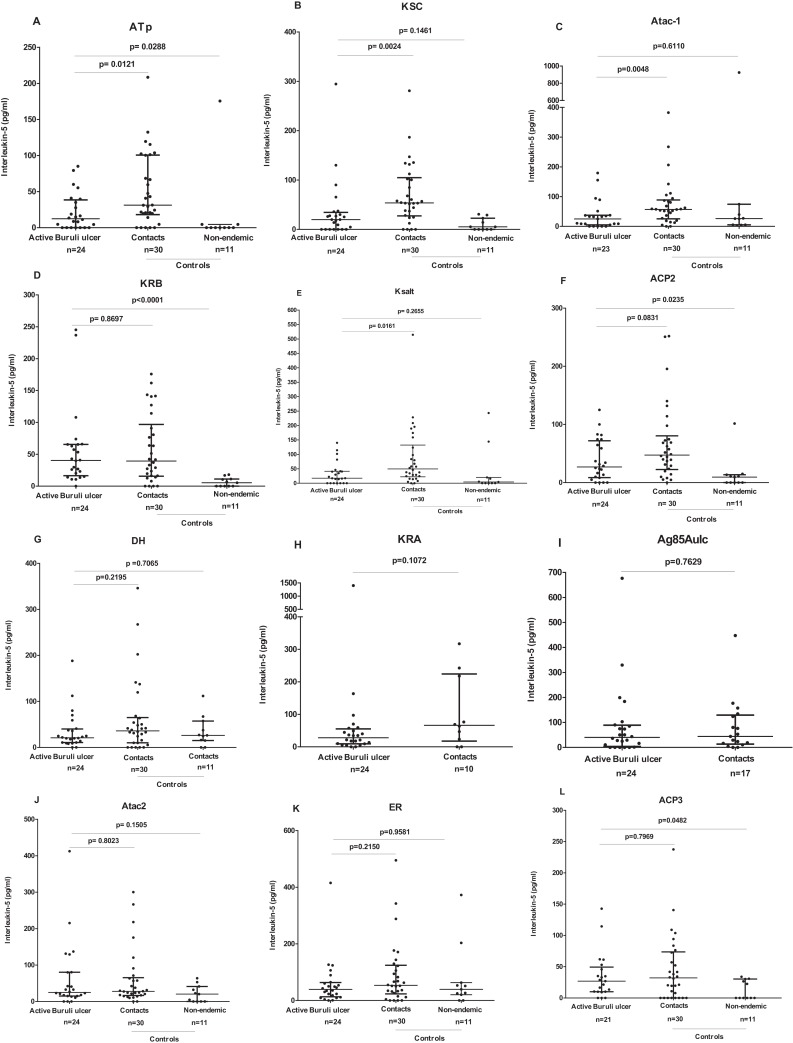
IL-5 responses to a panel of polyketide synthase domain antigens in 5 day diluted whole blood culture supernatants of *Mycobacterium ulcerans* disease patients, healthy endemic controls and healthy non-endemic controls. IFN-γ responses are shown for ATp (A), KSC (B), Atac1 (C), KRB (D), Ksalt (E), ACP2 (F), DH (G), KRA (H), Ag85Aulc (I), Atac2 (J), ER (K) and ACP3, (L). Each dot represents the response of one study participant. The horizontal lines represent the median and Interquartile range for each group. Medians for groups were compared using Mann-Whitney *U-test*.

When individual responses were compared, contacts of Buruli ulcer patients produced significantly higher IFN-γ responses compared with those of patients for ATp (median 3,416 vs 449 pg/ml), Ksalt (4,611 vs 521 pg/ml), ACP2 (3,794 vs 319 pg/ml), KS C (3,945 vs 618 pg/ml), DH (median 4,596 vs 671 pg/ml), ER (5,024 vs 3,010 pg/ml), KR B (5,261 vs 1,004 pg/ml), Atac2 (4,900 vs 1,215 pg/ml) (*P* < 0.05). Despite a trend to higher IFN-γ responses in contacts compared with cases it did not reach significance for ATac1, ACP3, KR A and *M. ulcerans* Ag 85A (Fig. 3). Similarly, comparing IL-5 responses (albeit low), contacts of Buruli ulcer patients produced significantly higher IL-5 responses compared with those of patients for ATp (median 31 vs 12 pg/ml), KS C (54 vs 20 pg/ml), Atac1 (57 vs 25 pg/ml), (*P* < 0.05). There were no significantly higher IL-5 responses in contacts compared with cases for KR B, DH, ACP2, ER, ACP3, KR A, Atac2 or *M. ulcerans* Ag85A (Fig. 4).

There were no significant differences in IFN-γ and IL-5 responses when patients with ulcerative disease were compared with those with non-ulcerated (earlier) forms of disease (Figs. S1 and S2).

### Comparison of IFN-γ responses of Buruli ulcer patients before and after treatment

**Figure 5:**
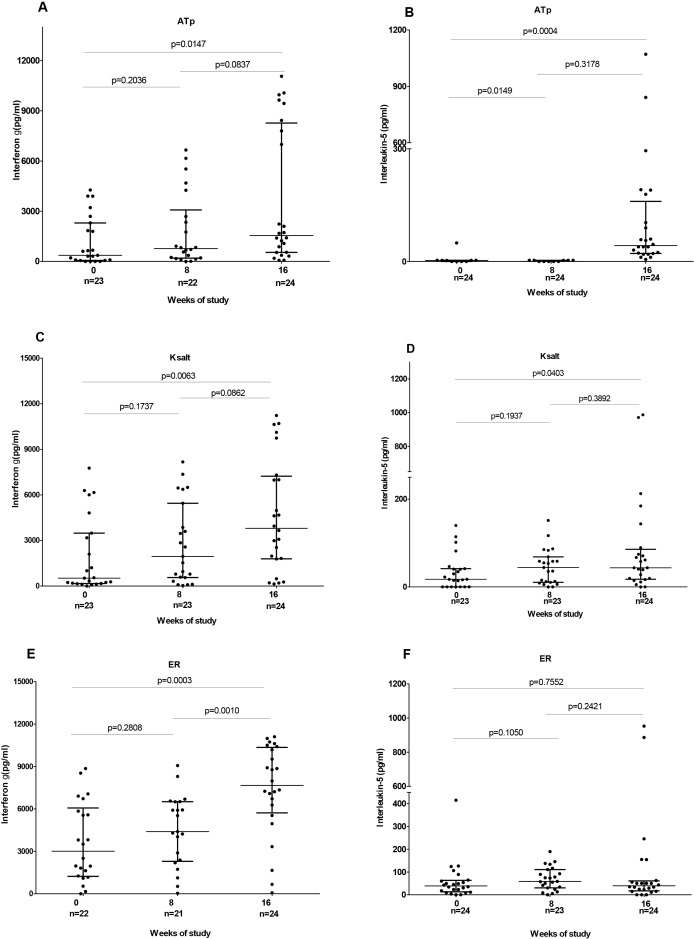
IFN-γ and IL-5 responses before (week 0), at completion of antibiotic treatment (week 8) and 8 weeks after antibiotic completion (week 16) to polyketide synthase antigens. IFN-γ responses are shown for ATp (A), Ksalt (C) and ER (E) while IL-5 responses are shown for ATp (B), Ksalt (D) and ER (E). Comparison between groups was done with Mann-Whitney test. Each dot represents the response of one study participant. The horizontal lines represent the median and Interquartile range for each group.

Significantly higher responses to recombinant antigens ACP2 and ATac2 (*P* < 0.05) were observed after 8 weeks standard antibiotic treatment compared with those at baseline. There was no significant difference in response to the other candidate antigens before and after 8 weeks treatment (Table 2). Responses to three of the most immunogenic antigens were also tested at week 16, 8 weeks after completion of treatment and were found to be significantly higher for ATp (1,544 vs 363.6, *P* = 0.015), Ksalt (3,803 vs 521.2, *P* = 0.006) and ER (7,662 vs 3,010, *P* = 0.0003) than at baseline. Further, IFN-γ responses to ER at week 16 were significantly higher than those at week 8 (7,662 vs 4,397, *P* = 0.001) (Fig. 5).

**Table 2:** IFN-γ and IL-5 responses to a panel of PKS antigens and Antigen85A after diluted whole blood stimulation for 5 days of 24 *Mycobacterium ulcerans* disease cases.

Cytokines [median(range) pg/ml)]	Antigens	Week 0	Week 8	*P*-value^‡^	Week 16	*P*-value^‡‡^
Interferon-γ	ACP2	319.1 (0–6,939)	1,004 (13–6,584)	0.0462	ND	
	ACP3	33.55 (0–2,054)	1 (0–3,507)	0.3881	ND	
	ATac1	1,270 (0–7,753)	28,439 (226–8,776)	0.1108	ND	
	ATac2	1,215 (0–7,753)	4,973 (0–12,355)	0.0494	ND	
	ATp	449.4 (0–4,269)	770 (0–6,659)	0.2036	1,544 (61–11,066)	0.0294
	ER	3,010 (0–8,855)	4,397 (38–9,064)	0.2808	7,662 (64–11,103)	0.0003
	KR A	2,850 (0–8,797)	3,561 (4–11,288)	0.3887	ND	
	KR B	1,004 (0–7,299)	4,851 (25–10,745)	0.0568	ND	
	KS C	618.4 (0–7,277)	1,190 (25–8,101)	0.2725	ND	
	Ksalt	521.2 (0–7,764)	1,950 (37–8,173)	0.2166	3,803 (136–11,229)	0.0169
	DH	671.2 (0–6,587)	2,037 (44–9,086)	0.077	ND	
	Ag85Aulc	307.4 (0–3,440)	2,287 (0–6,735)	0.0551	ND	
Interleukin-5	ACP2	26.94 (0–125.1)	52.87 (0–178)	0.1013	ND	
	ACP3	24.19 (0–114.4)	45.46 (0–1,396)	0.2089	ND	
	ATac1	25.02 (0–179.5)	52.7 (0–286.2)	0.0129	ND	
	ATac2	23.22 (0–412.4)	47.46 (0–167.3)	0.9695	ND	
	ATp	12.4 (0–85.08)	42.55 (0–144.6)	0.0149	42.35 (5.59–1,071)	0.002
	ER	39.14 (0–415.4)	58.82 (0–189.6)	0.105	39.47 (0–952.4)	0.2511
	KR A	28.07 (0–1,401)	46.34 (0–124.5)	0.1039	ND	
	KR B	40.44 (0–245.3)	42.58 (0–148.5)	0.5197	ND	
	KS C	19.84 (0–294.6)	36.00 (0–149.7)	0.1284	ND	
	Ksalt	17.37 (0–140)	44.19 (0–151.5)	0.1937	43.51 (0–987.1)	0.1067
	DH	20.98 (0–188.2)	28.17 (0–162)	0.4519	ND	
	Ag85Aulc	40.06 (0–676.7)	51.9 (0–614.9)	0.2236	ND	

**Notes:**

ND, not done.

‡ Mann–Whitney test comparing baseline (week 0) IFN-γ and IL-5 responses with those of with week 8.

‡‡ Mann–Whitney test comparing baseline (week 0) IFN-γ and IL-5 responses with those of with week 16. *P* < 0.05 indicate a significant difference.

**Figure 6:**
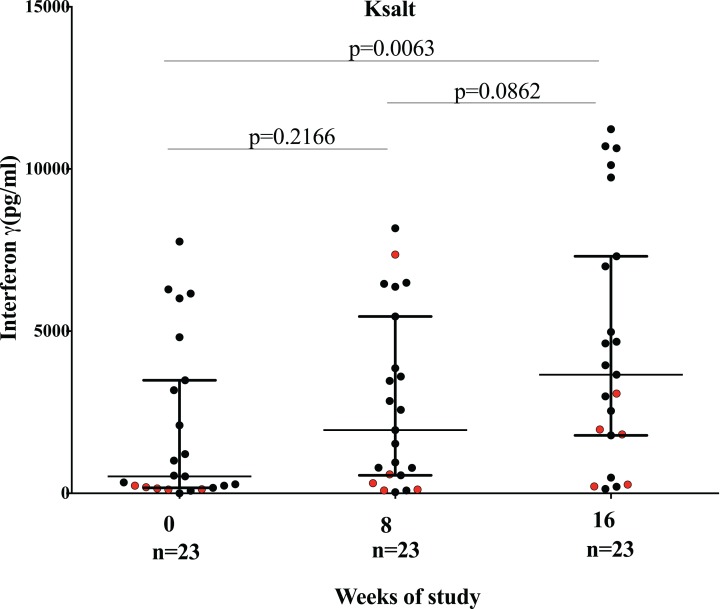
Comparison of IFN-γ responses before (week 0), at completion (week 8) of antibiotics and 8 weeks after antibiotic completion (week 16) to plasmid DNA encoding mycolactone PKS antigens Ksalt. Red dots represent IFN-γ responses of patients with detection of viable organisms at week 8. Each dot represent response for one study participant. The horizontal lines represent the median and Interquartile range for each group.

There was no correlation between clinical form of disease, duration of lesion, category of lesion, presence of BCG scar, time to healing and level of IFN-γ responses to Ksalt and ER antigen. However, patients with viable organisms at baseline and week 8 produced low IFN-γ responses to Ksalt with the exception of one patient that made a good response at week 8 (Fig. 6).

Significantly higher IL-5 responses to recombinant antigens ATac1 and ATp (*P* = 0.0129 and *P* = 0.0149 respectively) were observed after 8 weeks standard antibiotic treatment. There was no significant difference in response to the other PKS antigens before and after 8 weeks treatment (Table 2). There was no correlation between clinical form of disease, duration of lesion, category of lesion, time to healing and level of IL-5 responses but patients with BCG scars had significantly higher levels for Ksalt (*P* = 0.0108) and ACP2 (*P* = 0.0160) compared to patients who had no BCG scars (Figs. S3 and S4).

The three antigens that induced high IFN-γ responses were tested also for IL-5 production at week 16, 8 weeks after completion of treatment. ATp stimulated significantly higher IL-5 production [42.35 (5.59–1,071) *P* = 0.002] than at baseline but Ksalt and ER yielded no significant responses.

## Discussion

The immune response to *M. ulcerans* antigens observed in patients with Buruli ulcer and their contacts may give an indication of the responses desirable to protect against the disease. We have shown previously that whole blood IFN-γ responses to *M. ulcerans* sonicate were low in untreated patients but improved after treatment with antibiotics, and also that significant responses were mounted by contacts from endemic areas but not by people from non-endemic areas (Phillips et al., 2006; Sarfo et al., 2009). A unique characteristic of *M. ulcerans* is that it produces the toxin mycolactone which not only causes the cell necrosis associated with ulcers but is also immunosuppressive at low concentrations. Therefore, it is highly relevant to establish whether an immune response can be mounted to mycolactone. The molecule itself is lipid-like, being formed of a lactone ring and two polyketide side chains, so not surprisingly it is of low antigenicity (Dangy et al., 2016). However, it has already been shown that an IgG antibody response can be mounted to proteins generated by several PKS domains both in patients with Buruli ulcer and in their close contacts (Pidot et al., 2010). In the present study, we have investigated the cellular immune response to recombinant proteins representing 11 PKS enzymatic domains and to recombinant Ag85A of *M. ulcerans* in Buruli ulcer patients and their healthy contacts as well as in people from non-endemic areas. All the proteins tested induced IFN-γ secretion in the whole blood stimulation assay, the strongest response being associated with ER and Ksalt. IL-5 responses were more modest, and at best only 54% of patients responded to KR B and 73% of contacts to KS C, suggesting that the major type of response generated in vivo is TH-1. It is of considerable interest that the highest responses were seen in contacts of patients rather than in patients themselves. There are two possible explanations for this. The most likely explanation is that ongoing mycolactone production suppresses the immune response of patients but a predisposition to reduced immune responsiveness must also be considered. Against this is the fact that IFN-γ responses to ATp, Ksalt and ER improved significantly after antibiotic treatment for 8 weeks and, in the case of ER, had continued to increase after 16 weeks. At non-cytotoxic concentrations, mycolactone displays immunomodulatory properties on human primary monocytes and dendritic cells, indicating that the toxin may limit the triggering of innate immune responses in vivo (Coutanceau et al., 2007; Simmonds et al., 2009). By blocking the capacity of primary T-cells to produce multiple cytokines upon activation and by impairing T-cell migration and homing into lymph nodes, mycolactone can significantly inhibit the development of the adaptive immune responses to *M. ulcerans* (Boulkroun et al., 2010; Guenin-Mace et al., 2011). More recent evidence has also revealed that mycolactone exerts a profound effect on protein secretion by blocking the co-translational translation of a plethora of proteins such as TNF and IL-6 as well as nearly all other proteins that pass through the endoplasmic reticulum for secretion or placement in cell membranes (Hall & Simmonds, 2014; Ogbechi et al., 2015).

It has been hypothesized that a significant proportion of the population residing in an area of *M. ulcerans* endemicity may have been exposed to *M. ulcerans* without developing disease (Gooding et al., 2002). Our results support this concept and may also give an indication of the most important antigens for providing protection, ATp, Ksalt and ER being possible candidates. This could be tested in an animal model and in earlier studies it was found that mice vaccinated with ATp, ER and KRA showed a modest reduction in bacterial load or prolonged survival following *M. ulcerans* challenge, albeit to a lower extent than mice vaccinated with Ag85A or *M. bovis* BCG (Roupie et al., 2014). In another model of mouse infection with *M. ulcerans*, IFN-γ knockout mice displayed faster disease progression compared to wild type mice (Bibert et al., 2017). This supports the existing paradigm that IFN-γ production by T-cells is a key marker for host defence against this mycobacterial infection.

## Conclusion

We have demonstrated strong human cytokine responses specific for PKS domains in healthy contacts of Buruli ulcer patients, with lower responses in patients which improved after antibiotic treatment. ER and load module ketosynthase domain (Ksalt) were the most immunogenic antigens for IFN-γ responses, whereas KS C and KR B were the most immunogenic for contacts and cases, respectively, with regard to IL-5 production. A vaccine made up of the most immunogenic mycolactone PKS domains for TH-1 response (IFN-γ) combined with the mycolyltransferase Ag85A warrants further study.

## Supplemental Information

10.7717/peerj.5294/supp-1 Supplemental Information 1 Fig. S1. Dot plots comparing the median interferon gamma response of patients with non-ulcerative lesions versus ulcerative lesions to specific PKS domain antigens after diluted whole blood stimulation. Each dot represents the response of one patient. Cytokine response (IFN-g) on the Y-axis and the type of lesion presented (Clinical form) on the X-axis. The horizontal lines represent the median and Interquartile range for each group. Medians were compared using Mann-Whitney U test.

10.7717/peerj.5294/supp-2 Supplemental Information 2 Fig. S2. Dot plots comparing the median interleukin-5 response of patients with non-ulcerative lesions versus ulcerative lesions to specific PKS domain antigens after diluted whole blood stimulation. Each dot represents the response of one patient. Cytokine response (IL-5) on the Y-axis and the type of lesion presented (Clinical form) on the X-axis. The horizontal lines represent the median and Interquartile range for each group. Medians were compared using Mann-Whitney U test.

10.7717/peerj.5294/supp-3 Supplemental Information 3 Fig. S3. Dot plots comparing the median interferon gamma response of patients with BCG scar versus No BCG scar to specific PKS domain antigens after diluted whole blood stimulation for 5 days. Each dot represents the response of one patient. Cytokine response (IFN-g) on the Y-axis and the indication of effective BCG vaccination (BCG scar) on the X-axis. The horizontal lines represent the median and Interquartile range for each group. Medians were compared using Mann-Whitney U test.

10.7717/peerj.5294/supp-4 Supplemental Information 4 Fig. S4. Dot plots comparing the median interleukin -5 response of patients with BCG scar versus No BCG scar to specific PKS domain antigens after diluted whole blood stimulation for 5 days. Each dot represents the response of one patient. Cytokine response (IL-5) on the Y-axis and the indication of effective BCG vaccination (BCG scar) on the X-axis. The horizontal lines represent the median and Interquartile range for each group. Medians were compared using Mann-Whitney U test.

10.7717/peerj.5294/supp-5 Supplemental Information 5 Raw data for the study.

## Additional Information and Declarations

### Data Availability

The following information was supplied regarding data availability: The raw data are provided in the Supplemental File.
