# Supplementary figures and images for: IFN-γ and IL-5 whole blood response directed against mycolactone polyketide synthase domains in patients with Mycobacterium ulcerans infection
Source: PeerJ. 2018 Jul 31;6:e5294. doi: 10.7717/peerj.5294 (PMC6078848; doi:10.7717/peerj.5294)

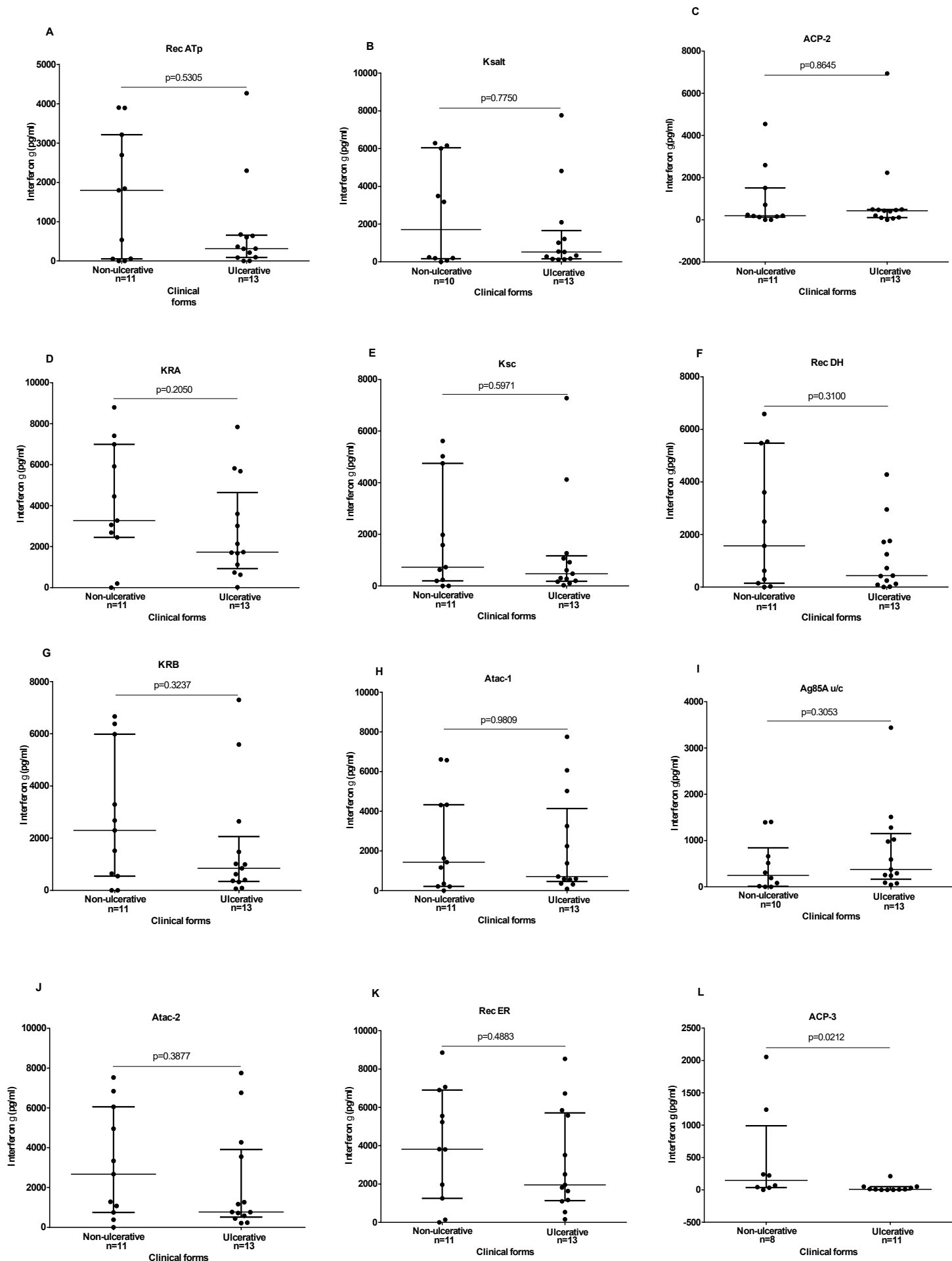

Supplement: Supplemental Information 1 — Each dot represents the response of one patient. Cytokine response (IFN-g) on the Y-axis and the type of lesion presented (Clinical form) on the X-axis. The horizontal lines represent the median and Interquartile range for each group. Medians were compared using Mann-Whitney U test. [file peerj-06-5294-s001.pdf]

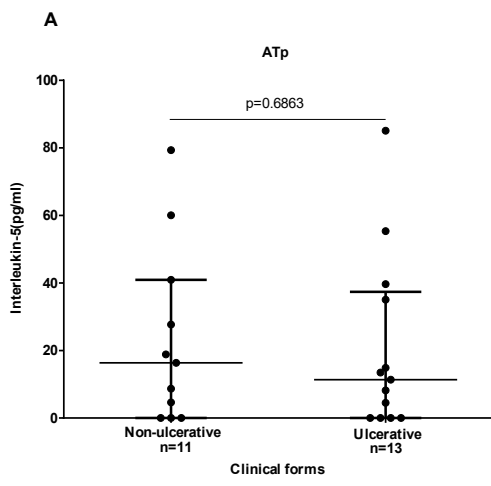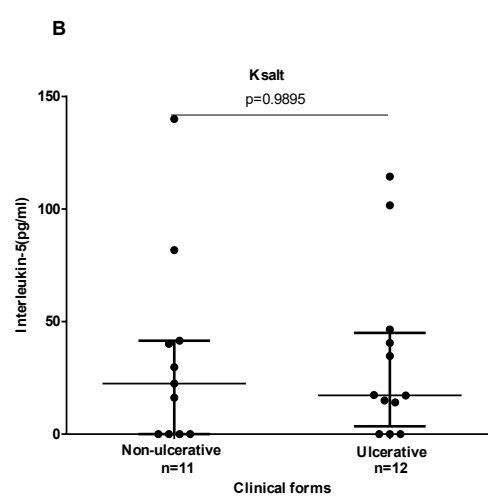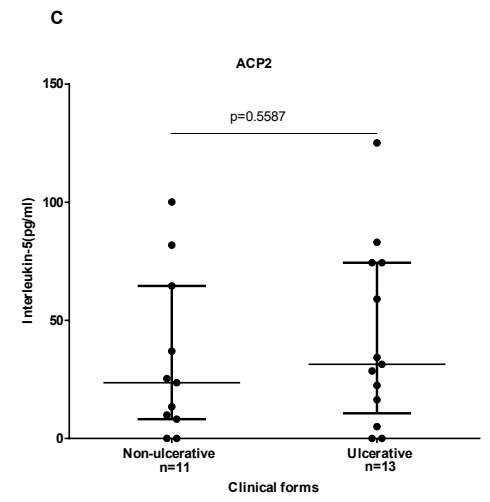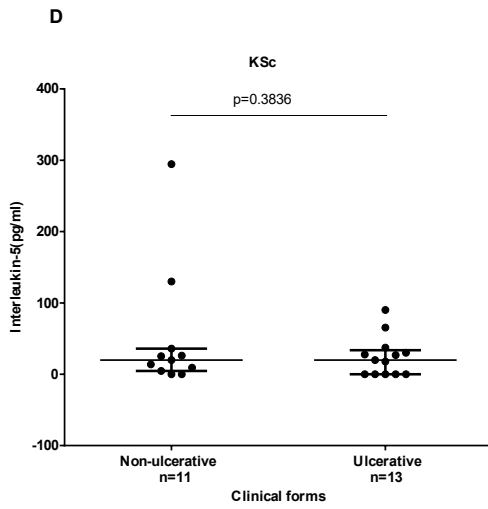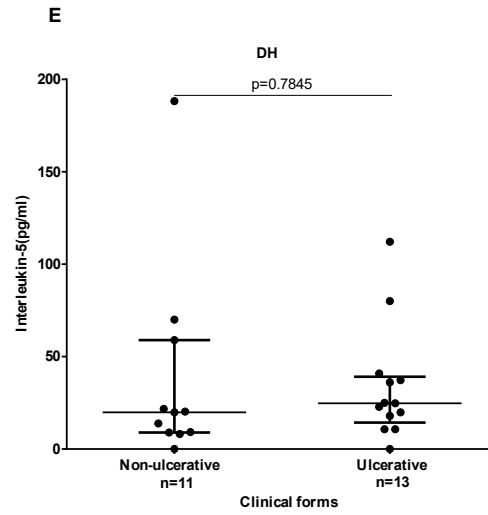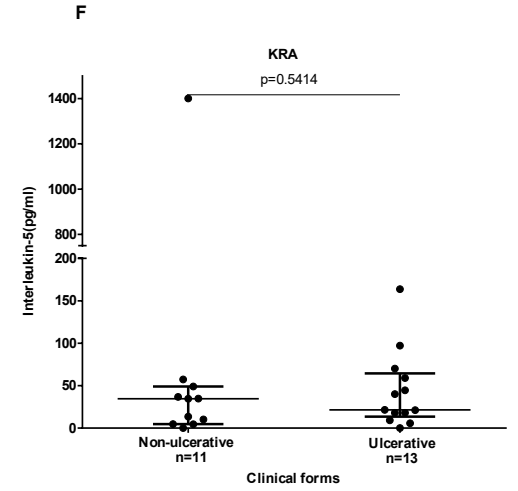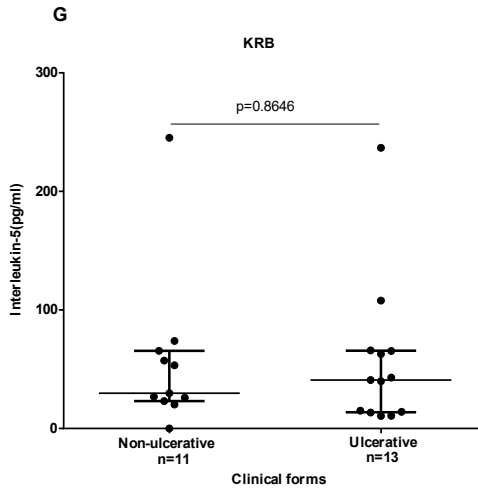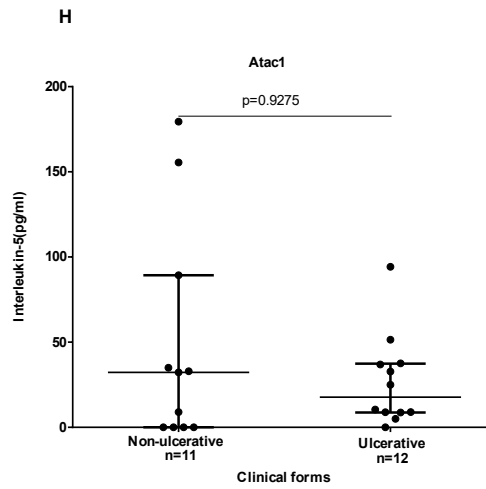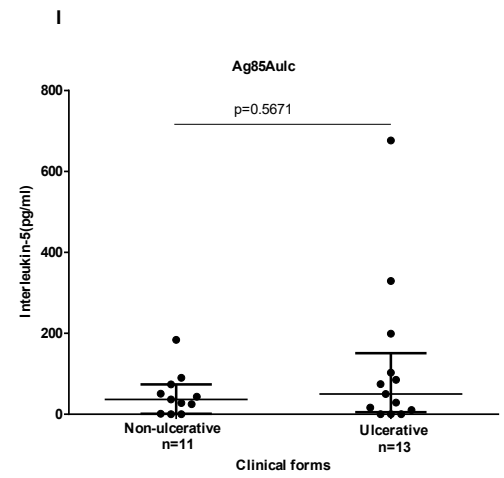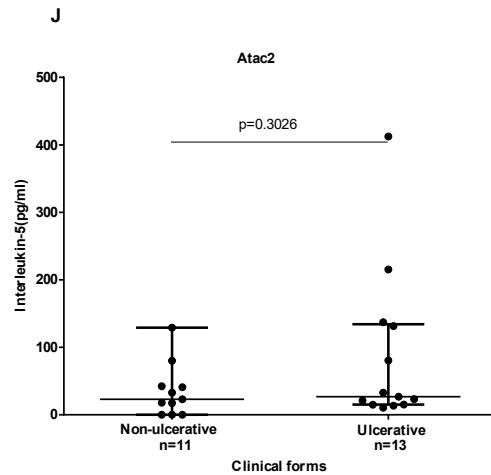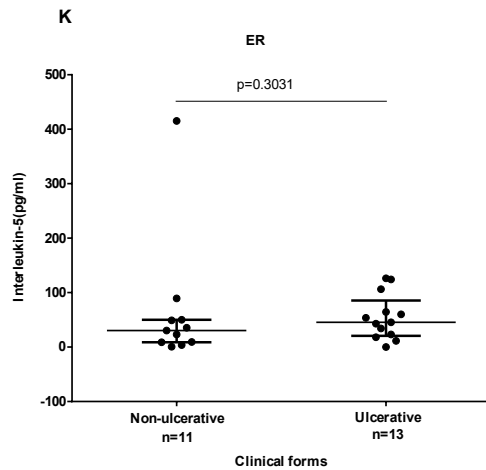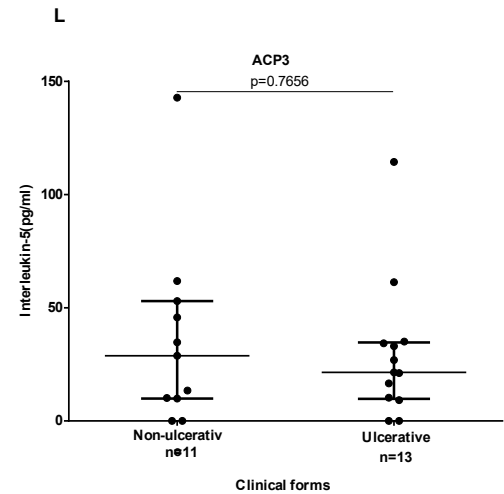

Supplement: Supplemental Information 2 — Each dot represents the response of one patient. Cytokine response (IL-5) on the Y-axis and the type of lesion presented (Clinical form) on the X-axis. The horizontal lines represent the median and Interquartile range for each group. Medians were compared using Mann-Whitney U test. [file peerj-06-5294-s002.pdf]

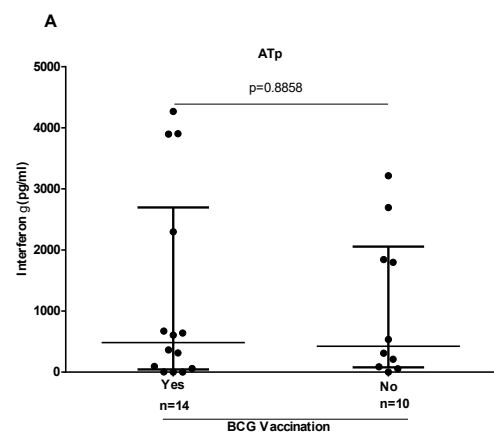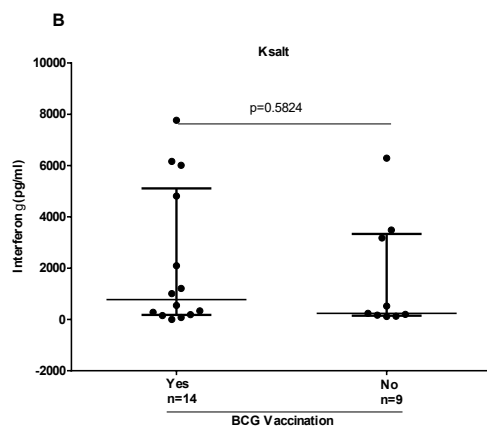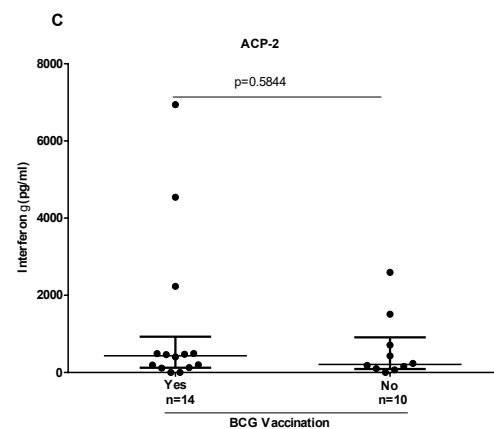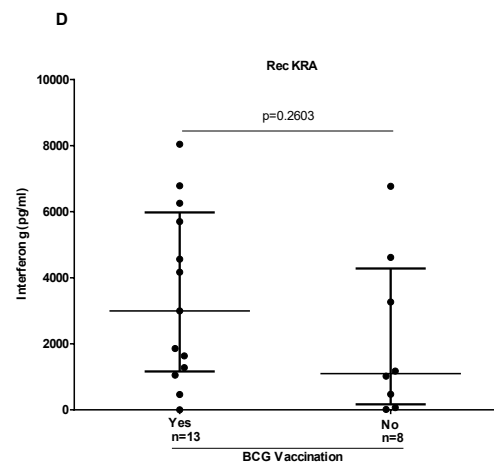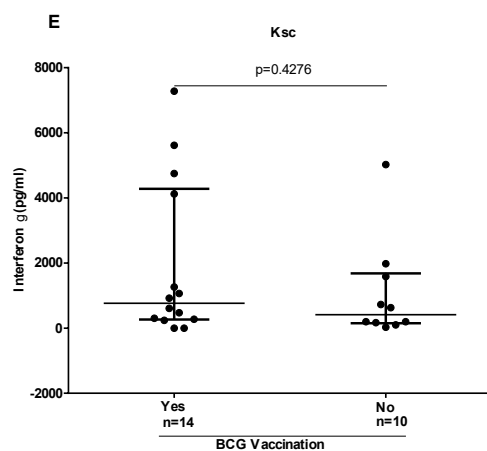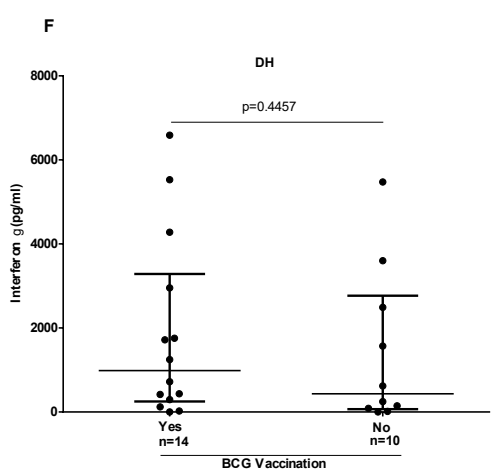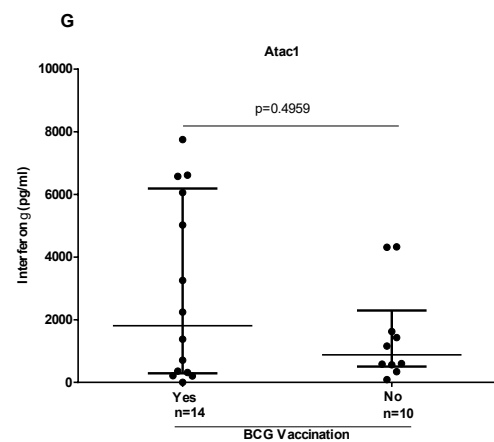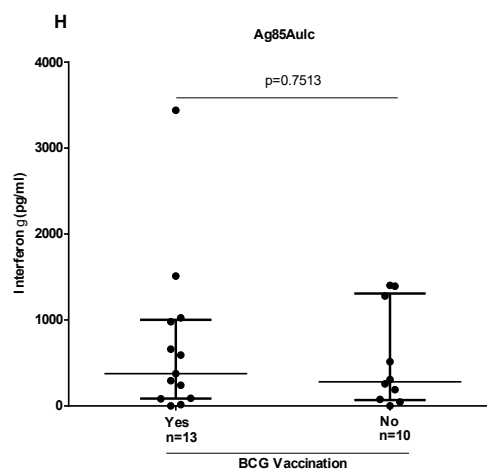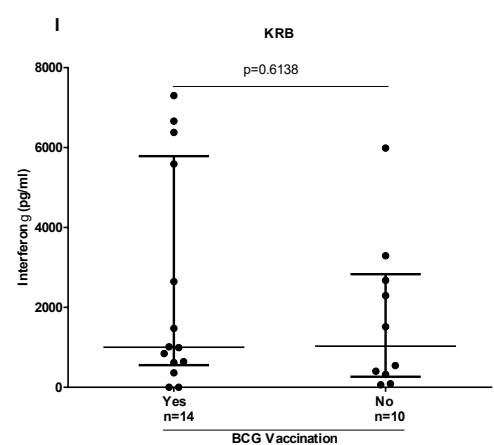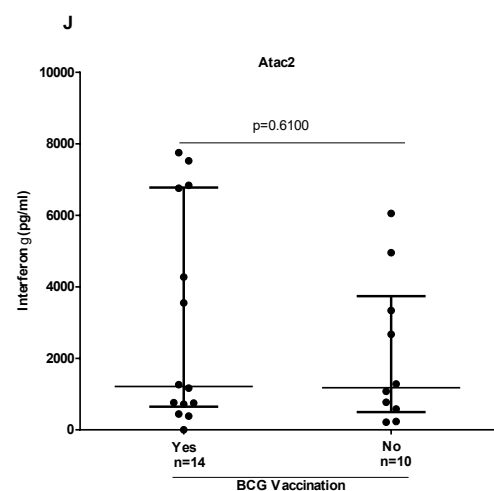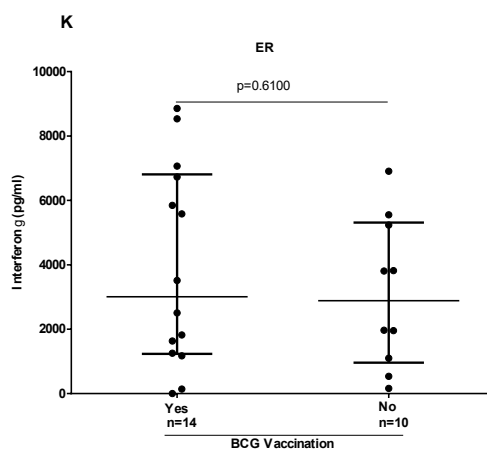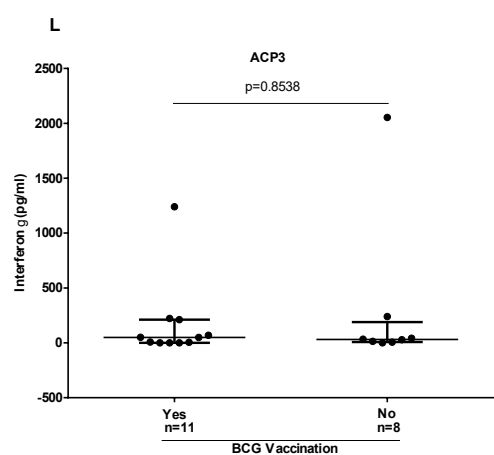

Supplement: Supplemental Information 3 — Each dot represents the response of one patient. Cytokine response (IFN-g) on the Y-axis and the indication of effective BCG vaccination (BCG scar) on the X-axis. The horizontal lines represent the median and Interquartile range for each group. Medians were compared using Mann-Whitney U test. [file peerj-06-5294-s003.pdf]

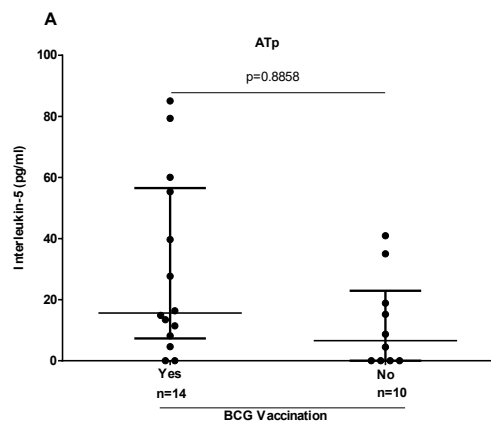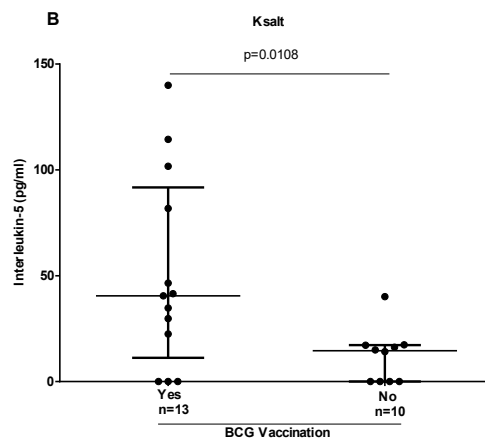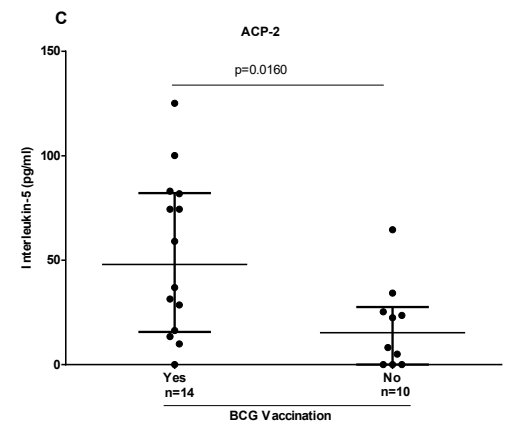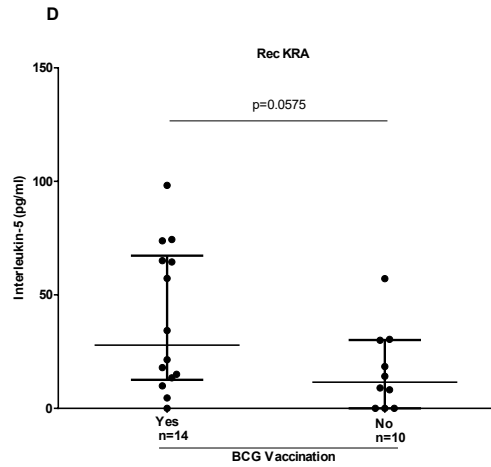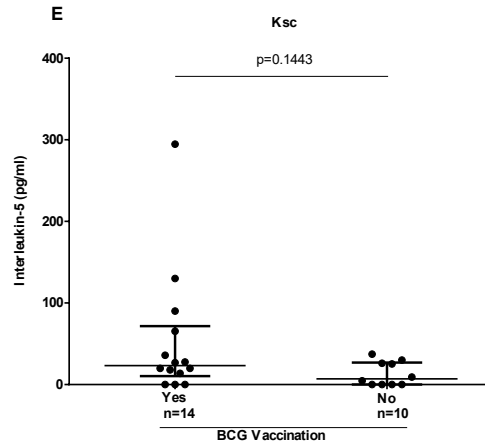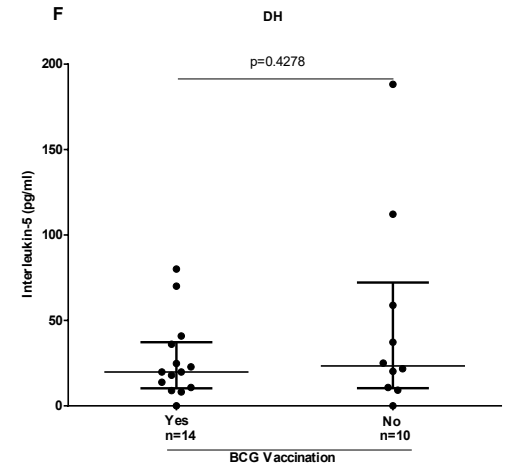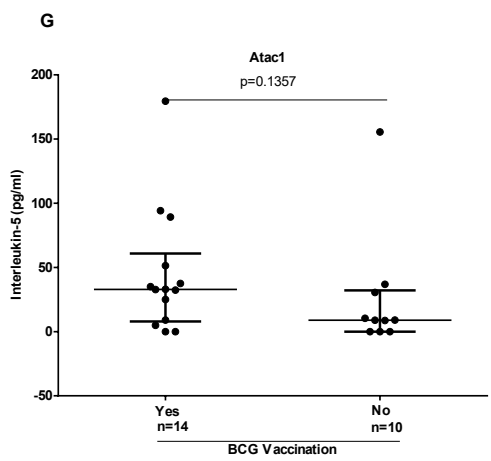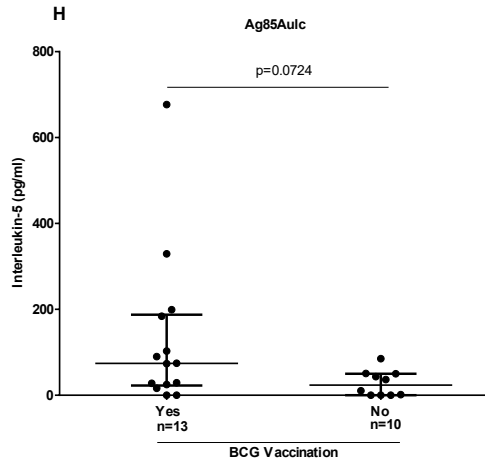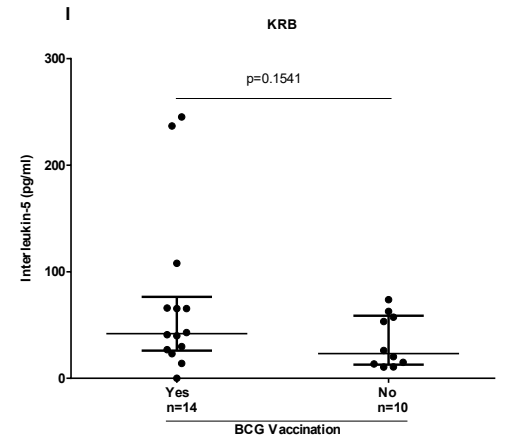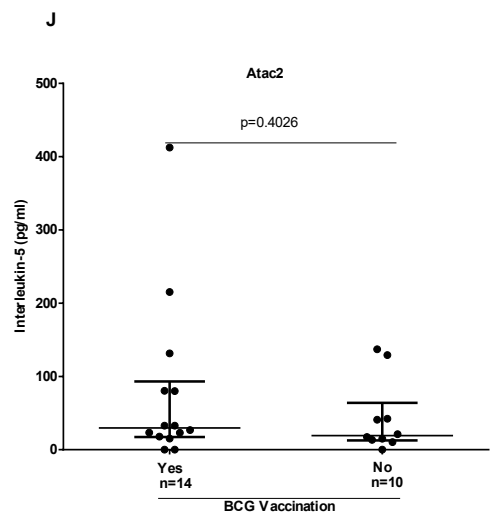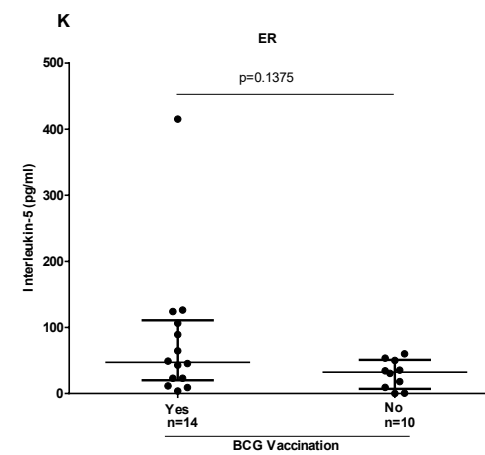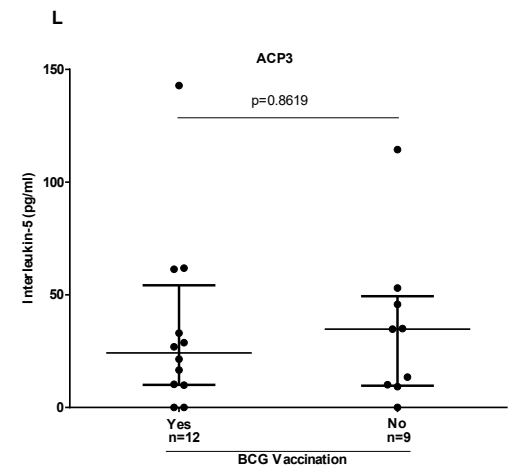

Supplement: Supplemental Information 4 — Each dot represents the response of one patient. Cytokine response (IL-5) on the Y-axis and the indication of effective BCG vaccination (BCG scar) on the X-axis. The horizontal lines represent the median and Interquartile range for each group. Medians were compared using Mann-Whitney U test. [file peerj-06-5294-s004.pdf]
